# 4096 Refined structure of human ferroportin using restraints from mass spectrometry

**DOI:** 10.1017/cts.2020.314

**Published:** 2020-07-29

**Authors:** Christian S. Parry, Andrey Ivanov, Guelaguetza Vazquez-meves, Fatemah A. Alhakami, Jessika Agyepong, Kyungreem Han, Bernard R. Brooks, Sergei Nekhai

**Affiliations:** 1 Georgetown - Howard Universities; 2 Howard University; 3 NHLBI Laboratory of Computational Biology, NHLBI/NIH; 4 Laboratory of Computational Biology, NHLBI/NIH

## Abstract

OBJECTIVES/GOALS: Mammals require iron for hemoglobin, respiration, immunity and as cofactor in enzymes. But free iron is toxic from the production of reactive oxygen species. Ferroportin is the sole exporter of cellular iron and it crucially determines cellular and systemic iron levels. Labile iron must be tightly regulated. This requires structural understanding. METHODS/STUDY POPULATION: We built structure of human ferroportin (FPN1) using the ab ignition prediction approaches of Rosetta/Robetta and by comparative modeling with distance restraints in MODELLER. Templates selected were from solute carrier protein families of distantly related orthologs and homologs including a proton coupled peptide transporter (PDB ID: 4IKV) and the bacterial iron transporter in outward-open and inward-open states, (PDB ID: 5AYM, 5AYO). Each model was validated by experimental mass spectrometry data. The energy minimized structural model was inserted into a lipid bilayer, placed in a rectangular simulation box, covered with TIP3P water solvent balanced with counterions and conditioned. Finally, we carried out 350 nanoseconds molecular dynamics simulations. RESULTS/ANTICIPATED RESULTS: Our first model of FPN1 (571aa), using Rosetta/Robetta *ab initio* approach, resembles the structure of the proton-dependent transporter, POT and consists of 12 transmembrane helices. The membrane spanning helices veer away from the orientation in the structure of 4IKV. The alternate model using MODELLER and the method of satisfaction of constraints, returned one template, the structure of *Bdellovibrio bacteriovorus* iron (Fe^2+^) transporter homolog (5AYN, 440aa) with sequence identity of 19%. Aligning FPN1 on the template sequence incorporating structural information revealed better conservation (29%). This model also comprises 12 transmembrane helices in two bundles separated by a large intracellular loop. The iron binding site predicted in both models match the structures of distant bacterial homologs. DISCUSSION/SIGNIFICANCE OF IMPACT: We are using these experimentally verified structures and functional data to answer questions about the mechanism of ferroportin iron transport, structural dynamics and the significance of mutations in ferroportin seen in different populations, especially the Q248H mutation found in Africans and black Americans with moderate to high prevalence.

